# Assessing everyday action in young adult athletes using the Virtual Kitchen Challenge: Relations with conventional cognitive tests

**DOI:** 10.1017/S135561772510101X

**Published:** 2025-06

**Authors:** Rachel E. Mis, Taisei Ando, Takehiko Yamaguchi, Caroline Brough, Leah Michalski, Linda J. Hoffman, Ingrid R. Olson, Tania Giovannetti

**Affiliations:** 1 Department of Psychology and Neuroscience, Temple University, Philadelphia, PA, USA; 2 Applied Information Engineering, Suwa University of Science, Nagano, Japan

**Keywords:** College athletes, executive function, episodic memory, cognition, task performance and analysis, goals

## Abstract

**Objective:**

The ability to efficiently complete everyday tasks was evaluated with a novel, performance-based test called the Virtual Kitchen Challenge (VKC) in college athletes. Analyses focused on the effect of practice and associations between the VKC and conventional measures of cognition.

**Method:**

81 college athletes with and without self-reported concussion completed conventional cognitive tests and the VKC, a nonimmersive virtual-reality task that requires manipulating virtual objects on a touch screen to prepare a breakfast and lunch under two conditions: 1) Training condition with feedback and 2) Test condition without feedback. VKC performance was scored for completion time, percent of time working on-screen, number of interactions with target and distractor objects. Paired t-tests compared VKC Training and Test conditions, correlations examined relations between VKC performance and cognitive tests.

**Results:**

VKC performance was significantly better after practice, as noted by faster completion time, fewer screen interactions, and a higher proportion of time spent on-screen during Test vs. Training conditions. Interactions with distractors were too infrequent for analyses. Correlations showed VKC Training was associated with episodic memory abilities whereas VKC Test scores were associated with executive function. VKC scores did not differ between participants with versus without concussion.

**Conclusions:**

The VKC is a promising portable performance-based measure of subtle functional difficulties for young, high-functioning participants. The VKC automated scoring makes it highly efficient for large studies and clinical settings.

## Statement of Research Significance

### Research Question(s) or Topic(s):

The Virtual Kitchen Challenge (VKC), a novel computerized, performance-based test of everyday function was evaluated as a sensitive measure of subtle difficulties in young, high-functioning adults. Findings were interpreted from a cognitive processing framework called the goal-control model. **Main Findings:** Results showed everyday task performance improved with practice, and consistent with the goal-control model, performance was associated with episodic memory abilities when task/goal representations were relatively weak and less familiar, but after practice, performance was associated with executive function abilities. **Study Contributions:** The VKC yields automated, sensitive, and theoretically informed scores of everyday task performance in high-functioning adults that may be useful for large studies and clinical settings.

## Introduction

One important goal of neuropsychological assessment is to understand how cognitive abilities and difficulties might influence everyday functioning and to make recommendations to improve and optimize functioning. Neuropsychological assessment relies on objective, performance-based tests of cognition that have strong psychometric properties. However, the predictive validity or generalizability of results from traditional neuropsychological tests to real world functioning is limited (Mcalister et al., 2016; Royall et al., 2007), partly because traditional neuropsychological measures were not developed specifically to evaluate everyday function (Harvey, 2012). Implications of neuropsychological assessment for everyday function are indirect and must be inferred. Over the years, neuropsychologists have attempted to address this gap by applying the traditional approach to neuropsychological assessment (i.e., use of psychometrically strong, standardized, performance-based tests) to the direct evaluation and quantification of everyday, functional abilities (Chilton & Schmitter-Edgecombe, 2023; Schmitter-Edgecombe et al., 2012; Schwartz et al., 2002; Shallice & Burgess, 1991; Woods et al., 2017). Performance-based tests of everyday function have focused largely on clinical populations with moderate to severe cognitive impairments, as most performance-based tests are too easy for populations with mild difficulties (i.e., ceiling effects). The focus of this study was to extend the development of a novel performance-based test of function originally designed to be sensitive to mild difficulties in older adults (VKC; Giovannetti et al., 2019) for use in younger populations. We used the VKC and a cognitive processing model of everyday activities (Goal-Control Model) to learn more about everyday functioning in a sample of college students with and without a lifetime history of concussion.

Independent everyday functioning requires competence with a wide array of tasks. We focus our investigation on a domain of tasks that we call everyday activities, which include three defining features: 1) pursuit of a practical task goal, 2) serial ordering of task steps; and 3) selection and use of objects (Giovannetti et al., 2021). We consider the cognitive operations necessary for accurate and efficient performance of everyday tasks from a cognitive activation framework. Everyday activities are represented as schema hierarchies, and activation within the schema hierarchy may spread automatically from higher level schema (e.g., make lunch) to lower level schema (e.g., spread peanut butter) as well as from objects or environments that may “trigger” associated schema (e.g., a coffee mug may activate coffee-making schema; Reason 1980, Norman 1981, Cooper & Shallice, 2000, 2006). When interpreted from this framework, data from performance-based tests with participants with moderate level cognitive impairment have suggested that successful and error-free everyday task performance depends on intact **goal representations** of the overarching task (e.g., make breakfast) and subordinate subtasks (e.g., make coffee, add sugar, and so on) as well as executive **control** over the activation of relevant task goals, transitions to subtasks, and suppression of irrelevant goals (Goal-Control Model). The strength and integrity of goal representations improve with repeated practice and is dependent on the temporal lobe and associated cognitive processes, including semantic knowledge and episodic memory. By contrast, efficient control over goal representations is dependent on executive control processes and integrity of white matter tracts in older adults (Bailey et al., 2013; Brennan et al., 2009; Giovannetti et al., 2008, 2015, 2021; Seidel et al., 2013). The utility of the Goal-Control model for understanding everyday action in younger individuals with no to mild cognitive difficulties has not been explored, in part due to a lack of sensitive measures for assessing everyday task performance.

There are different approaches to increasing the difficulty and sensitivity of performance-based tests, including increasing the task complexity (Giovannetti et al., 2007; Schmitter-Edgecombe et al., 2012; Shallice & Burgess, 1991) or modifying the scoring/outcome measures (Divers et al., 2021; Rycroft et al., 2018a; 2018b; Seligman et al., 2014). We opted for the latter approach in our development of the VKC, a nonimmersive VR task that possesses all the defining features of everyday tasks in a simulated kitchen environment (Giovannetti et al., 2019). The VKC was based on validation work with a version that required use of a computer mouse to move objects and demonstrated strong correspondence with real tasks in people with dementia (Allain et al., 2014). Our version of the VKC uses a touch screen to make responses more naturalistic. Our VKC also yields sensitive measures of task efficiency, such as completion time, number of screen interactions required to complete the task, proportion of total time spent moving objects on the screen (vs. time off-screen thinking/planning next move). After brief training trials, participants are asked to complete breakfast and lunch test trials. Scores on the training and test trials have been validated in studies of healthy, community dwelling older adults against real task performance, traditional cognitive measures (Giovannetti et al., 2019), and neuroimaging (Holmqvist et al., 2024). Consistent with the Goal-Control Model, VKC performance improved with practice and was associated with measures of executive function, episodic memory, and white matter disease on brain MRI in older adults (Giovannetti et al., 2007; Holmqvist et al., 2024). Although the VKC has potential for understanding everyday function in clinical populations of all ages, including younger people with traumatic brain injury, severe mental illness, and other developmental and acquired disorders, to our knowledge the VKC has not been studied in younger adult participants (<65 years).

### Current study

The present study sought to extend the application of the VKC to younger adults. The validity of the VKC was examined in a well characterized sample of young adult athletes with and without a self-reported lifetime history of concussion (Hoffman et al., 2023). A prior publication of this sample focused on conventional neuropsychological tests, questionnaires, and neuroimaging of cerebral white matter, showed no differences on cognitive tests or measures of cerebral white matter tract-based spatial statistics in participants with a history of concussion versus participants with a history of one or more concussions, consistent with the larger neuropsychological literature on concussion suggesting time-limited, acute effects on cognition (Gaudet et al., 2022; Karr et al., 2014). In fact, studies suggest that those who experience persisting, long-term symptoms following concussion often have a premorbid history of conditions affecting cognition and function (Goreth & Palokas, 2019). Analyses in the current study focused on 1) examining differences in VKC training versus VKC test trials in the full sample; 2) identifying associations between VKC scores and traditional measures of cognition in the full sample. Based on results with older adults that showed practice improved measures of completion and planning/deliberation time but not the number of screen interactions, we hypothesized that younger adult participants would also show faster performance with practice. We next hypothesized that VKC completion time would be associated with cognitive tests of processing speed and that, consistent with the Goal-Control Model (Giovannetti et al., 2021), VKC Training and Test trials would differentially be associated with measures of episodic memory and executive functioning. Specifically, we reasoned that training trials, during which goal representations are relatively weaker would be more strongly associated with episodic memory abilities, whereas once the goal representations are more strongly established, performance on test trials would be more strongly associated with measures of executive control. Analyses of differences on the VKC between participants with vs. without a history of concussion were not the focus but were included in Supplementary Materials; all results showed no differences between concussion groups.

## Method

### Participants

Participants aged 18–40 were recruited from a parent study at Temple University examining the relation between sports-related concussion and substance use disorders in collegiate athletes (State of Pennsylvania, Department of Health CURE grant: “Mechanisms and treatment strategies to counter addiction susceptibility post TBI”). Participants were recruited into the parent study from the current and former Temple University student population and club sports in the Philadelphia community. A subset of these participants was then selected to undergo neuroimaging and additional testing (neuroimaging arm) and also comprised the sample for the present study. Exclusion criteria for the neuroimaging arm included current or past neurologic disorder or major psychiatric illness (e.g., epilepsy, brain tumor, schizophrenia, and bipolar disorder), current (within past four weeks) use of psychotropic medications, severe sensory/motor deficits that would preclude completion of study tasks, and contraindication for MRI scanning. Recruitment procedures and level of sport engagement are described in further detail in a prior publication (Hoffman et al., 2023).

### Procedures

Study procedures were approved by the Temple University Institutional Review Board, adhered to all university guidelines and the Helsinki Declaration, and included written informed consent from all participants. Participants completed the study in multiple visits. Demographics, concussion history, and other measures relevant to the parent study were completed during an initial visit. A second visit was completed by a subset of participants who consented to undergo neuroimaging and additional cognitive testing (neuroimaging arm of the parent study). Participants who enrolled in the neuroimaging arm completed cognitive testing and questionnaires in a single visit lasting approximately 1.5 hours after which they underwent brain MRI lasting one hour at the same session or a follow-up visit. Cognitive and questionnaire measures for the present study were administered by the lead author or trained research assistants.

### Virtual kitchen challenge (VKC)

**Figure 1.**
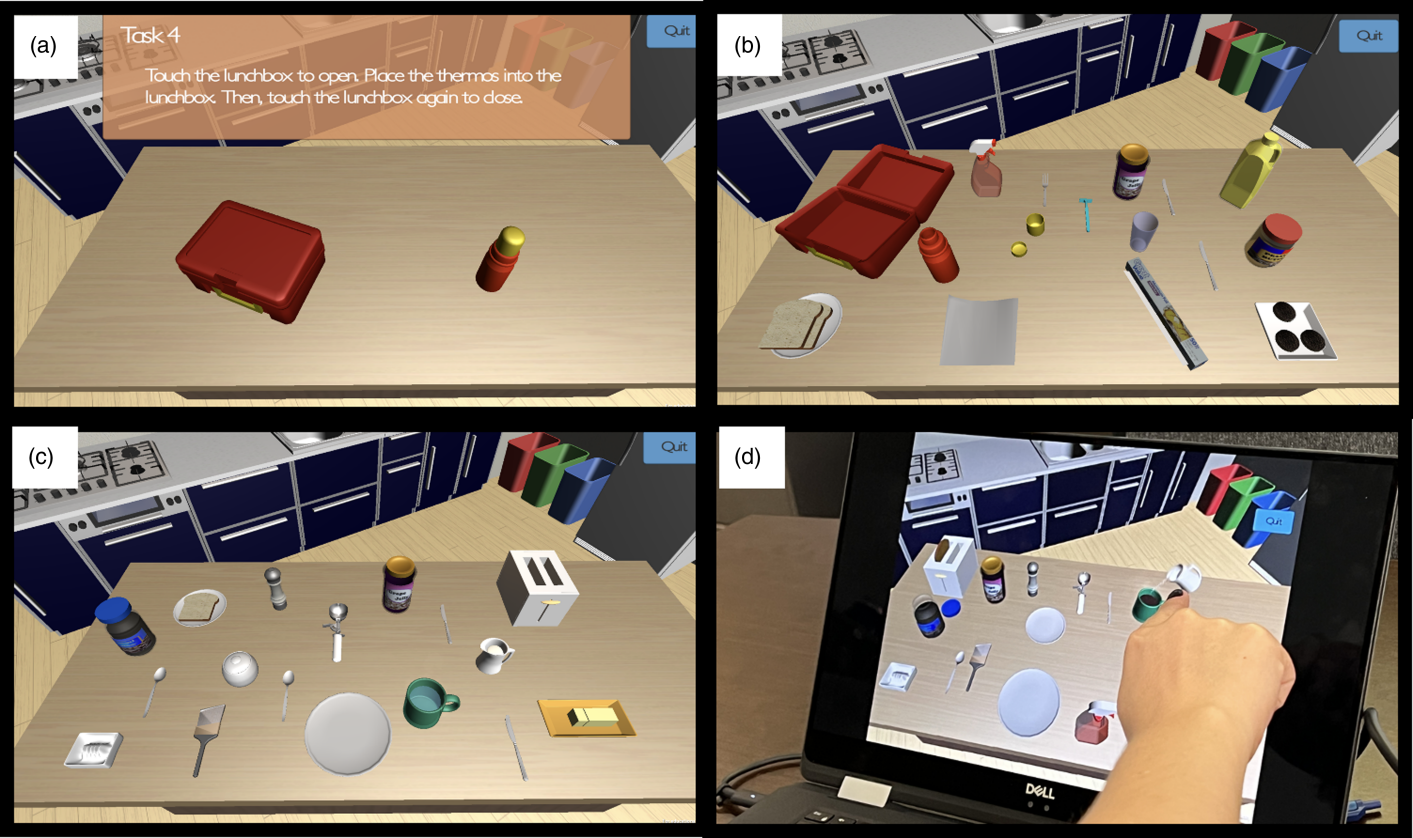
The virtual kitchen challenge a) screenshot of the basic training task used for familiarizing participants with the basic actions required to manipulate objects on the touchscreen; b) screenshot of the lunch task objects used for both the training and test conditions; c) screenshot of the breakfast task objects used for both the training and test conditions; d) photograph of a participant interacting with the computer touchscreen to complete the breakfast task.

Detailed information regarding VKC task administration and scoring have been published previously, along with preliminary analyses supporting the construct validity of the VKC scores in older adults (Giovannetti et al., 2019; Holmqvist et al., 2024). Briefly, the VKC is a nonimmersive, virtual reality task in which participants are asked to complete two everyday tasks of comparable complexity on a touchscreen: preparing a breakfast and a lunch. For each subtask, 10 target objects necessary for task completion and four distractor objects are presented on the screen as shown in Figure 1. Participants are instructed to use their dominant hand to manipulate objects to complete the tasks. Administration of the VKC consists of basic movement familiarization (unscored; Figure 1, panel A), a training condition on the breakfast and lunch, and the test condition on the breakfast and lunch. The order of the breakfast and lunch subtasks was counterbalanced across participants. A Dell XPS 15 9575 with Intel(R) Core (TM) i7-8705G CPU processor @ 3.10 Hz laptop computer with touchscreen was used to administer the VKC.

#### VKC training condition

After completing the movement familiarization sequence, participants completed a training condition for the breakfast/lunch trials. During training, the assessor first provided detailed task instructions (e.g., “Prepare a breakfast for someone who wants toast and coffee. This person wants a single slice of toast with butter and jelly and a cup of instant coffee with cream and sugar”) and verbally named and pointed to all target and distractor objects. Participants were allowed to ask any questions regarding the objects or task before proceeding. The assessor then provided the detailed task instructions a second time before participants were allowed to complete the training condition. Participants were permitted to ask questions during completion of the training condition, and the assessor provided immediate feedback on any errors.

### VKC test condition

The test condition for the breakfast/lunch were administered immediately following the training condition. Participants were provided with the task instructions as provided in the training condition. In addition, they were told to complete the task as quickly as possible without error and not to touch or move objects on the screen until they were ready to use them. The testing condition was completed in silence without assessor feedback.

### VKC automated scoring

Four automated scores were computed separately for the VKC training and test conditions. Completion time was calculated as the amount of time in seconds between the first screen interaction and pressing quit. Number of target interactions consists of the number of times a participant touched or moved any target object, whereas number of distractor interactions consists of the number of times a participant touched or moved any distractor object. Last, proportion of time on-screen was computed as the amount of time during which the participant was touching/moving objects on the touchscreen divided by total completion time. Preliminary studies in older adults suggest that longer completion times, higher number of target and distractor interactions, and lower proportion of time on-screen reflect less efficient performance on the VKC (Giovannetti et al., 2019; Holmqvist et al., 2024).

### Cognitive testing

Processing speed was assessed using the written trial of the Symbol-Digit Modalities Test (SDMT; Smith, 1982), and episodic memory was assessed with the Hopkins Verbal Learning Test – Revised (HVLT-R; Brandt, 1991). Both tests were administered and scored according to the manuals. The outcome for the SMDT was raw total correct, and the outcome for the HVLT-R was raw number of correct responses on the delayed recall trial. Tests of executive functioning consisted of the flanker, set-shifting, and N-back tasks from the National Institute of Health – Executive Abilities: Measures and Instruments for Neurobehavioral Evaluation and Research (NIH-EXAMINER) battery, which was administered and scored according to the manual (Kramer et al., 2014). As such, the outcome for the flanker was a composite score ranging from 0 to 10 reflecting both accuracy and speed of performance, with higher scores reflecting better performance; the outcome for the set-shifting task was computed in an identical manner. The outcome for the N-back task was the discriminability index (d prime) between the hit rate and the false positive rate. Finally, scores from the HVLT-R recognition trial were used to estimate performance validity, as past studies have suggested a cutoff of <5 for the HVLT-R Discriminability Index (Bailey et al., 2018; Sawyer et al., 2017). The psychometric properties of these measures are strong and have been reported in the test manuals (Brandt, 1991; Kramer et al., 2014; Smith, 1982).

### Analyses

All analyses were performed in IBM SPSS (version 29). Paired sample *t* tests examined differences between VKC training and test conditions on VKC automated scores. Correlation coefficients examined relations between VKC performance and cognitive test scores. Analysis of Variance (ANOVA) was used to compare VKC performance among participants who reported no history of concussion, a history of 1 – 2 concussions, or a history of 3 or more concussions (see Supplement).

## Results

### Participant characteristics, missing data, and data distributions

A total of 108 participants were enrolled in the neuroimaging arm of the parent study. An exclusionary neurologic abnormality was identified in one participant after MRI, and this participant was excluded from further data analyses. Additionally, due to a change in the VKC administration protocol, the first 26 participants enrolled in the study did not receive the VKC training condition and thus were excluded from analysis in this study. The final sample for the present study consisted of 81 participants. Participant demographics and mean scores on cognitive tests are depicted in Table 1. As shown in Table 1, on average participants were close to ceiling on the HVLT-R Recognition Discriminability Index and all participants scored well above the suggested cut-score of 5, suggesting adequate effort and valid performance scores.

**Table 1.** Descriptive characteristics for sociodemographic, concussion history, neuropsychological, and VKC performance variables for the study sample (*N* = 81)

	*M* (SD) / % of sample	Range
***Sociodemographic variables***		
Age (years)	21.63 (3.54)	18 – 33
Sex assigned at birth (% female)	51.90	–
Race (%)		
*Asian*	7.41%	–
*Black/African-American*	8.64%	–
*Native Hawaiian or Other Pacific Islander*	1.23%	–
*White/Caucasian*	74.07%	–
*Unknown*	2.47%	–
*More than 1 race*	6.17%	–
Ethnicity (%)		
*Hispanic or Latino*	8.64%	–
*Not Hispanic or Latino*	87.65%	–
*Unknown or do not wish to report*	3.70%	–
***Concussion history***		
Number of lifetime concussions	1.41 (1.47)	0 – 6
Concussion group membership		
*No lifetime concussions*	27%	–
*1-2 lifetime concussions*	38%	–
*3+ lifetime concussions*	16%	–
***Cognitive test scores***		
HVLT-R delayed recall	9.99 (1.88)	4 – 12
DSMT	61.21 (9.91)	38 – 88
Flanker	9.02 (.45)	7.74 – 9.75
Shifting	9.00 (.54)	7.77 – 10.00
n-back	1.66 (.72)	.33 – 3.39
HVLT-R Recognition Discriminability	11.37 (.96)	7 – 12
HVLT-R Recognition Discriminability ≥5	100%	

HVLT-R = Hopkins Verbal Learning Test-Revised; DSMT = Digit Symbol Modality Test

Data were excluded and/or missing for one participant on the Digit Symbol Modality Test (DSMT) due to failure to follow task instructions, for two participants on the 2-back task due to inability to complete the practice trials (one participant) and computer error (one participant), and for one participant on the flanker due to computer error. All other variables were complete for all participants.

Data were normally distributed for VKC training and test proportion of time on-screen, DSMT, shifting, and *n*-back. Values outside two standard deviations from the mean were winsorized for VKC training completion time (four values), VKC training total interactions (four values), VKC test completion time (three values), VKC test total interactions (five values), and flanker (two values). HVLT-R delayed recall performance was negatively skewed, and thus nonparametric tests are reported for analyses involving this variable.

### VKC training vs. test trials

Scores on the VKC Training and Test Trials are reported in Table 2. Number of distractor interactions was at floor for both the training and test conditions, with only one participant interacting with distractor object(s) for the training condition and five participants interacting with distractor object(s) for the test condition, and thus this variable was not included in statistical analyses. For all other VKC scores, paired sample *t* tests revealed that participants performed significantly better on the test condition than the training condition. Specifically, participants were significantly faster (Total Completion Time), spent a larger proportion of time on-screen, and had fewer total number of screen interactions (Number of Target Interactions) when performing the VKC test condition.

**Table 2.** VKC performance variables from the Training and Test Conditions (N = 81)

	Training condition		Test condition		Paired sample *t*-tests (df = 80)		
	*M* (SD)	Range	*M* (SD)	Range	*t*	*p*	*d*
Total completion time (sec)	153.46 (22.38)	111.40 –208.00	113.34 (15.36)	82.42 – 149.00	14.46	< .001	1.61
*M* %time on-screen	47.28 (8.46)	27.90 –63.05	52.27 (9.15)	31.72 – 67.43	5.30	< .001	0.59
Target interactions	49.84 (10.92)	34 –77	40.16 (8.17)	28 – 59	9.06	< .001	1.01
Distractor interactions	.01 (.11)	0 –1	.07 (.31)	0 – 2	–	–	–

### VKC performance and cognition

Relations between VKC performance and cognitive test scores are reported in Table 3. As predicted, performance on the VKC Training condition was associated with episodic memory abilities. Specifically, better HVLT-R delayed recall performance was associated with fewer total screen interactions for the VKC training (*ρ* = −.31, *p* = .005) but not the test condition. Also as predicted, performance on the VKC Test condition was associated with executive function abilities, but so was performance on the VKC Training condition. Specifically, better flanker performance was associated with a higher percentage of time on-screen during the test condition (*r* = .25, *p* = .03) and fewer total interactions during the training condition (*r* = −.33, *p* = .003). Finally, the test of processing speed was associated with completion time only for the VKC training condition, as better DSMT performance was associated with faster completion times on the VKC training condition (*r* = −.25, *p* = .02) but not the test condition. All other associations were not statistically significant.

**Table 3.** Correlation coefficients between VKC performance and cognition (N = 81)

	Episodic memory HVLT-R r (p)	Processing speed DSMT r (p)	EF/Inhibition (Flanker) r (p)	EF/Shifting (Shifting) r (p)	EF/Working memory (*n*-Back) r (p)
Training Completion Time	−.15 (.19)	−.25 (.02)*	−.12 (.27)	−.02 (.85)	−.16 (.17)
Training Percent Time On-Screen	−.02 (.90)	−.08 (.49)	.17 (.14)	−.04 (.74)	.17 (.14)
Training Total Interactions	−.31 (.005)**	−.21 (.06)	−.33 (.003)**	−.16 (.17)	−.12 (.29)
Test Completion Time	.08 (.47)	−.15 (.17)	−.18 (.11)	−.10 (.37)	.00 (.99)
Test Percent Time On-Screen	−.08 (.46)	−.13 (.25)	.25 (.03)*	.11 (.33)	.03 (.81)
Test Total Interactions	−.09 (.44)	.04 (.73)	−.15 (.20)	−.15 (.17)	−.11 (.32)

HVLT-R= Hopkins Verbal Learning Test-Revised; DSMT = Digit Symbol Modality Test; EF = executive function

Note: Bold indicates statistically significant correlation coefficients. Spearman’s correlations are reported for Episodic Memory HVLT-R associations with VKC performance, all others are Pearson’s correlations.

*p < .05; **p < .01

## Discussion

A novel performance-based test of function, called the VKC, which has been validated as a measure of mild functional difficulties in older adults, was administered to college student athletes with and without a lifetime history of concussion. The goal of the study was to extend our work with the VKC and learn more about everyday function in young adults. Results showed significant improvement in everyday task speed and efficiency after practice, as noted by faster completion time, fewer screen interactions to complete the task, and more time spent on-screen, rather than off-screen planning or mis-reaching. Training and test conditions also were associated with different cognitive abilities and generally supported predictions from the Goal-Control Model that Training trials would be associated with episodic memory abilities whereas Test trials would be associated with measures of executive function. Finally, VKC scores did not differ between participants with versus without a lifetime history of concussion (See Supplement), extending the extant literature on cognitive tests and neuroimaging and suggesting that self-reported lifetime history of concussion in college athletes does not significantly disrupt the capacity to accurately and efficiently perform real world tasks.

Participants showed significant improvement in all measures of VKC task speed and efficiency after the training condition, with moderate to large effect sizes. Benefits from the same training protocol were also observed for completion time and proportion of time on-screen in a previously published study of older adults with and without mild cognitive impairment. However, unlike the current sample of young adults, older adults did not show improvement on total screen interactions. For older adults, the average number of screen interactions remained at approximately 60 for both conditions (Holmqvist et al., 2024), whereas younger adults completed the VKC in approximately 50 interactions but dropped down to 40 interactions in the Test condition. Of note is that average scores on the Test condition from the young participants in this sample were comparable to a small sample of college students reported in a previous study (Giovannetti et al., 2019) and were not at ceiling, as the VKC may be completed in as few as 30 screen interactions by experts. Although practice effects may not be surprising, the everyday tasks included on the VKC are highly familiar and might have been expected to be performed at ceiling by young adults. Our results suggest that VKC automated measures are sensitive to change and may be useful to detect change in functional abilities in longitudinal studies, including clinical trials, for young adults with a range of cognitive ability levels.

Overall, the younger adults in this sample showed more efficient VKC performance on all measures (completion time, proportion on-screen, and interactions) compared to previously published scores from older adults (Giovannetti et al., 2019; Holmqvist et al., 2024). VKC differences between younger and older adults may be explained by differences in cognitive abilities, which are well established in the cognitive aging literature (Salthouse, 2004). Age differences also may be influenced by differences in computer/touchscreen experience, peripheral motor difficulties, computer self-efficacy/anxiety, and so on. Future work is needed to understand age effects on the VKC efficiency scores, but prior studies showing similar age effects on measures of subtle errors and task efficiency on the same breakfast and lunch tasks with real objects (Divers et al., 2021; Rycroft et al., 2018a) along with correlation analyses showing relations between VKC scores and tests of cognitive abilities, suggest age differences are at least partially due to differences in cognitive abilities.

Correlation analyses in the current sample of young adults showed significant relations between VKC scores and measures of cognitive abilities. As predicted on the Goal-Control Model, the measure of episodic memory was significantly associated with performance (i.e., number of interactions) only during the Training condition when representations of the task goals were relatively weak. Past studies of older adults with dementia have shown that measures of episodic memory abilities and medial temporal lobe structures are associated with omission errors on performance-based everyday tasks with real objects (Bailey et al., 2013; Giovannetti et al., 2008; Seidel et al., 2013). Such observations and others (Brennan et al., 2009; Giovannetti et al., 2007, 2015) informed the Goal-Control Model, linking the strength of task goal representations with episodic memory. The current results support this notion, as when participants were learning the tasks and the VKC goals were relatively weakly represented (VCK Training condition), performance was significantly associated with episodic memory skills, but, after task goals were strengthened through practice (VKC Test condition), relations with measures of episodic memory were weak and nonsignificant.

Predictions regarding cognitive correlates of the VKC Test condition were partially supported, as the executive function measure of inhibition (Flanker) was associated with the proportion of time spent on-screen during the Test condition. Specifically, participants with greater inhibitory control (i.e., higher score on the Flanker) spent more time on-screen versus off-screen. Based on findings from VKC validation studies, off-screen time is spent deliberating and mis-reaching toward the screen in error and is associated with weaker cognitive abilities and larger volumes of white matter hyperintensities in older adults (Giovannetti et al., 2019; Holmqvist et al., 2024). The Flanker score also was associated with the number of interactions during the VK Training condition, indicating that participants with greater inhibitory control were able to complete the VKC training tasks with fewer screen interactions, possibly because they resisted interference from competing off-task objects/actions.

As predicted, completion time was significantly associated with the conventional test of processing speed, but only for the training condition. Overall, the pattern of correlations showed stronger relations between cognitive scores and measures on the Training condition versus the Test condition. The pattern of correlations suggests that the Training condition, when the task is more novel and less familiar, may require cognitive abilities that are more similar to abilities measured by conventional tests. Future work is needed to determine whether performance on the VKC Training versus the Test conditions is more strongly associated with real life everyday function.

We acknowledge several limitations of the current study. First, the sample did not include clinical groups with known functional impairment and no measure of functional abilities of the sample against which to compare the VKC scores. However, there is no ground-truth measure of everyday function, as questionnaires are influenced by recall bias and mood and are often insensitive to mild difficulties. Future work on everyday function should consider use of ecological momentary assessment or passive sensing/digital phenotyping for everyday activity tracking to determine whether VKC scores predict real world function.

In conclusion, the VKC was designed to include the three defining features of everyday activities: 1) pursuit of a practical task goal, 2) serial ordering of task steps; and 3) selection and use of objects (Giovannetti et al., 2021). Our results support the VKC as a performance-based test of everyday function that is sensitive to change and subtle variations in cognition in a young, high-functioning sample. The virtual reality platform, which enables administration on a laptop computer with a touchscreen, support the portability of the VKC. The automated scoring derived from the touchscreen data supports the scalability of the VKC for large studies and busy clinical settings.

## Supporting information

Mis et al. supplementary material Mis et al. supplementary material

## Supplementary material

To view supplementary material for this article, please visit https://doi.org/10.1017/S135561772510101X.
