# Supplementary material for: Assessing everyday action in young adult athletes using the Virtual Kitchen Challenge: Relations with conventional cognitive tests
Source: J Int Neuropsychol Soc. 2025 Sep 1;31(5-6):423–9. doi: 10.1017/S135561772510101X (PMC12771397; doi:10.1017/S135561772510101X)
Supplement: Mis et al. supplementary material [file S135561772510101Xsup001.docx]

**Concussion History and Performance on the Virtual Kitchen Challenge**

Concussion history was determined using the Ohio State University Traumatic Brain Injury Identification Method (OSU TBI-ID), a structured interview for obtaining lifetime history of TBI (Corrigan & Bogner, 2007). As described in Hoffman et al. (2023), concussion was operationalized as any head injury in which a participant endorsed feeling dazed, experiencing a memory gap, or losing consciousness. Participants were grouped according to their total number of lifetime concussions.

**Results**

One-way ANOVA revealed no statistically significant differences in VKC performance based on lifetime concussion status (training completion time: *F*(2, 78) = .80, *p* = .45; training percent time on-screen: *F*(2, 78) = .21, *p* = .81; training total interactions; *F*(2, 78) = .51, *p* = .60; test completion time: *F*(2, 78) = 1.42 *p* = .25; test percent time on-screen: *F*(2, 78) = 2.21, *p* = .12; test total interactions: *F*(2, 78) = .84, *p* = .44). Post-hoc tests revealed no significant differences for any variables based on concussion history (all *p*’s > .13). There were also no statistically significant differences in neuropsychological performance for omnibus (HVLT delayed recall: *F*(2, 78) = 1.73, *p* = .18; DSMT: *F*(2, 77) = .16, *p* = .85; flanker: *F*(2, 77) = 2.81, *p* = .07; shifting: *F*(2, 78) = .48, *p* = .62; *n*-back: *F*(2, 76) = .82, *p* = .44) or post-hoc tests (all *p*’s > .05). Means and standard deviations for VKC and cognitive performance variables based on concussion history are included in **Table S1**.

| **Table S1.** VKC and Cognitive Performance by Concussion Group | | |  |
| --- | --- | --- | --- |
|  |  |  |  |
|  | M (SD) | | |
|  | No Lifetime Concussions | 1-2 Lifetime Concussions | 3+ Lifetime Concussions |
| ***VKC Performance, Training Condition*** |  |  |  |
| Total completion time (sec) | 151.64 (24.53) | 152.07 (17.66) | 159.80 (28.37) |
| Mean percent of time on-screen | 47.46 (9.14) | 46.71 (7.91) | 48.32 (8.96) |
| Number of target interactions | 51.33 (11.27) | 49.61 (11.65) | 47.88 (8.52) |
| ***VKC Performance, Test Condition*** |  |  |  |
| Total completion time (sec) | 110.44 (15.18) | 116.37 (16.40) | 111.03 (12.33) |
| Mean percent of time on-screen | 51.21 (8.94) | 51.23 (9.88) | 56.51 (6.59) |
| Number of target interactions | 41.56 (7.97) | 39.97 (8.54) | 38.25 (7.66) |
| ***Neuropsychological Functioning*** |  |  |  |
| HVLT-R delayed recall | 9.70 (2.02) | 9.87 (2.02) | 10.75 (1.00) |
| DSMT | 60.58 (8.09) | 61.16 (10.87) | 62.38 (10.70) |
| Flanker | 9.14 (.34) | 9.02 (.43) | 8.81 (.58) |
| Shifting | 9.08 (.49) | 8.98 (.57) | 8.92 (.58) |
| n-back | 1.76 (.71) | 1.67 (.76) | 1.47 (.64) |

HVLT-R = Hopkins Verbal Learning Test-Revised; DSMT = Digit Symbol Modality Test

**Conclusion**

Participants with versus without a lifetime history of concussion did not differ on any of the VKC scores in either the Training or Test conditions. There were no significant differences even when comparing participants with three or more concussions to those without a concussion history. Do the similarities among the concussion groups on the VKC suggest that the VKC is failing to detect subtle functional difficulties in those with a history of concussion, or that there is truly no effect of lifetime history of concussion on functional abilities? In our view all of the available evidence suggests the latter conclusion. Participants in this study with versus without a self-reported history of concussion also did not differ on conventional cognitive tests, including sensitive tests of processing speed and executive function abilities. In a prior publication that included the participants in the current study, concussion status also was not associated with any neuroimaging measure of white matter microstructure or white matter connectivity (Hoffman et al., 2023). Additionally, VKC measures were significantly correlated with measures of cognitive abilities in this sample and in prior studies of older and younger participants (Giovannetti et al., 2019; Holmqvist et al., 2024). Finally, VKC measures significantly correlated with measures of white matter hyperintensities in a group of older adults (Holmqvist et al., 2024). Thus, when considering all of the results from the present and prior validation work with the VKC, we conclude that self-reported history of past concussion in college students does not affect the capacity to accurately and efficiently perform real world tasks.  However, we acknowledge that our results do not generalize to the acute phase of concussion or to the minority of people who experience persisting cognitive impairment following concussion.

We acknowledge that the self-report measure of concussion was a limitation of this study. Self-report is prone to error due to recall failures and/or denial. Nevertheless, self-report is the method used in neuropsychological assessment and the OSU TBI-ID is the current gold standard self-report measure. As noted earlier, our results do not generalize to people in the acute phase of concussion recovery or to those with post-concussive syndrome.
